# Supplementary material for: Predicting partner fitness based on spatial structuring in a light-driven microbial community
Source: PLoS Comput Biol. 2023 May 3;19(5):e1011045. doi: 10.1371/journal.pcbi.1011045 (PMC10184905; doi:10.1371/journal.pcbi.1011045)
Supplement: Attachment — Submitted filename: Response to reviewers (6).docx [file pcbi.1011045.s005.docx]

Response to reviewers - *Predicting partner fitness based on spatial structuring in a light-driven microbial community*

We thank the reviewers for their thoughtful comments. Please see our in-line responses to suggestions and queries below (green text).

Reviewer's Responses to Questions

**Comments to the Authors:
Please note here if the review is uploaded as an attachment.**

**Reviewer #1:**

1. Line 171: Is it mu or mu_i?

We apologize for the confusion and thank the reviewer for their attention to detail. µ_i_  is appropriate for line 171, since the variable in question refers to the growth rate of an individual cell, not that of the population. We have modified the text accordingly.

2. Lines 186-189: For equations 5-8, psi is used instead of psi_i, is there a difference? If yes, what is the formula for psi?

Similarly to point 1, ψ_i_ is appropriate. We have modified the text to correct this error.

3. Line 282: Are there links to the custom models mentioned? Or sample NUFEB input files?

The modified NUFEB source code can be found at: <https://github.com/jsakkos/NUFEB>, as mentioned in our Data Availability statement on Lines 466-467. The custom models that were developed in this study have also been incorporated into the main NUFEB repository here: <https://github.com/nufeb/NUFEB>. Example input files can be found at <https://github.com/nufeb/NUFEB/tree/master/examples>. Alternatively, they can be generated with the Python helper package, nufeb-tools (<https://github.com/Jsakkos/nufeb_tools>), with the command *nufeb-seed*.

4. Line 305: Did you mean sucrose biomass accumulated?

The text is correct in that the rate of total biomass accumulation (sucrose + cell) increases after sucrose secretion machinery is expressed. When the sucrose-exporting S. elongatus strains were originally reported, this was an unexpected phenotype. However, this is now been characterized in greater detail in other published work, as referenced in the subsequent lines:

*“This phenomenon is likely due in part to increased overall photosynthetic flux and CO_2_ fixation rates in S. elongatus when these pathways are engaged [25,26], is assisted by documented increases in Rubisco abundance following sucrose export [55] and potentially, that the specific ATP/NADPH requirements may alter the balance of metabolite pools important for bioproduction [56,57].”*

5. Line 339: It is unclear how fig. 4B shows the negative correlation.

We apologize for the lack of clarity in the statement at Line 339. The negative correlation refers to the decrease in S. *elongatus* cell biomass with increasing sucrose secretion (IPTG). We have modified the text as follows to make the point clearer:

*As expected, the rate of sucrose export was strongly correlated with the rate of E. coli biomass accumulation (Figure 4A), while it was negatively correlated with S. elongatus cell biomass (Figure 2A-2B, 4B): greater sucrose export led to slower S. elongatus growth.*

**Reviewer #2:**

1. Parameters were obtained from experimental data but in different culture conditions (different media). This is problematic for the parameterization and the prediction of establishing a stable consortium. If these two strains have already proven to form a consortium, why not grow them in the same media culture?

The reviewer brings up a salient point. In the manuscript on lines 431-437 we addressed this as being part of future work:

*In order for experimental co-cultures to be grown that have comparable growth kinetics to the simulations, a bi-species optimized medium is required. Our growth models were fit to experimental data on species-specific media optimized for axenic growth (i.e., BG-11 and R/2). Previous studies have used modified BG-11, typically supplemented with additional sources of nitrogen [31,34]. While this does enable E. coli to grow, the density of their growth is more than an order of magnitude less than possible on rich media [36,38].*

To elaborate further on this question, there is still some uncertainty about the most appropriate medium to use under co-culture conditions. The main issue with the medium that we used previously to co-culture the two species is that the heterotroph cannot thrive in it. While BG-11 medium supplemented with ~ 4 mM NH_4_Cl (Hays *et. al.* 2017) does allow *E. coli* growth in the absence of any other carbon source, it does not allow *E. coli* to grow exponentially; instead, the cells are limited by nitrogen and other nutrients and do not exceed OD_600_ of ~ 1, even with excess carbon (*i.e.*, supplemented sucrose or glucose). Thus, when characterizing *E. coli* growth in BG-11 supplemented with 4 mM NH_4_Cl with the goal of predicting behaviors for co-culture, axenic *E. coli* are unable to utilize much of the secreted sucrose. R/2 medium, which we used in this study, has been previously shown to support *E. coli* W growth quite well (Arifin *et. al.* 2014).

R/2 and BG11 media have a great deal of similarities in their formulation. In a series of experiments conducted for work described in manuscript, but which we did not elaborate upon in this manuscript, we examined the primary components important for improved *E. coli* growth and found that the inclusion of yeast extract was the primary determinant of improved *E. coli* performance. Unfortunately, yeast extract is a complex component and its inclusion tends to stunt the growth of *S. elongatus* in R/2 medium. *E. coli* growth in co-culture experiments with a BG-11 base medium tends to out-perform axenic controls (see, for example, Hays *et al.*, 2017), most likely due to the secretion of other, complex metabolites by *S. elongatus*. Taken together, optimization of a medium that is compatible with both species and that minimizes .

2. The authors talk about the stability of natural consortia. As the simulations run to a steady state. It would have been interesting to subject the simulations to a change of environment, to challenge this stability. Can this be done on this platform?

The reviewer brings up a very interesting question and future line of inquiry. Indeed, perturbations to the environment (*e.g.*, nutrient concentrations, cell concentration, etc.) can be implemented with NUFEB. Our long-range goals for this work are focused upon pairing simulation datasets with experimental data generated from growth of co-cultures in microfluidic wells. As we implement this goal, it will become possible to do experiments where more control can be used in altering environmental conditions will positional elements of cells are maintained.

3. For measurements and validation of the IBM model, how many cells are in each experiment? 11 OD is a very high measurement; this could easily measure with a flow cytometer and correlated with the total number of cells in the computational simulation. These results could be relevant as the prediction from the model is that initial density is a determinant in the establishment of the consortia and also the accumulation of sucrose is also an important factor.

We did not specifically measure the number of cells during the experimental validation of the model. Based on OD_600_ to CFU/mL data from Hays *et. al.* 2017 (Figure S3), we would expect approximately 1100 *E. coli* W cells in a 100 x 100 x 10 µm chamber at OD_600_ 11. This estimate is within an order of magnitude of the actual cell numbers from our simulations.

4. If E coli utilize far more sucrose than S. elongatus can produce, do the authors obtain conditions by which there is competitive exclusion?

In the current study, we did not find conditions where there was competitive exclusion. For one, the two species were not competing for the same resource, aside from space. Moreover, *E. coli* cannot survive under the conditions examined in the absence of *S. elongatus* (or an alternate source of organic carbon).

5. I think the discussion could be improved by discussing how to integrate the findings in real experiments, as it has already been published that these two strains can be co-culture.

We thank the reviewer for this point. Beyond developing an optimal growth medium for both species, which was discussed in Lines 432-438, we have added Lines 439-445 discussing the experimental implementation:

*Experimental investigation of spatial effects between these two species will be required to further validate the NUFEB model and broaden the scope of its utility. Mimicking the growth chambers simulated in this work can be achieved either with isolated microwells, droplets [59], or microfluidic chambers [60,61], which have previously demonstrated support for cyanobacterial growth [62]. With timelapse microscopy for observation of microbial growth coupled with downstream image analysis [63,64], experimental efforts will be synergistic to complement NUFEB [65].*

6. A flask with constant shaking is usually used as a proximation of a homogeneous environment; why is it a spatial environment? Does any of these strains form spatially structured formations as biofilms? Consortia can survive in a homogenous environment, or if sucrose can diffuse further?

The reviewer brings up a good point: the conditions used to fit the growth models were substantially different than the conditions we simulated. We do not consider flasks to be spatial environments; they are approximately homogeneous, as the reviewer noted. However, growth in flasks has several advantages for gathering data to inform simulations. 1) Flask growth is homogenous and easily replicated. 2) Measurements of cell density and cell number over time are easily and accurately accomplished. Since our computational model takes into account the local concentration of nutrients, we can still fit the growth data from flasks into a spatial environment, so long as the spatial environment is not exceedingly nutrient limiting (for the fitting). In order to best reproduce the conditions in a flask, we used the exact same starting cell densities and did not allow the cultures to overgrow (Figures 2 and 3).

*S. elongatus* does not form biofilms, but it is likely that *E. coli* W would, though it is unclear whether that would be possible under the conditions studied here (*i.e.* maximal cell density, incubation time).

It is unclear what the reviewer is asking in the last question, but we take it to ask whether the consortia can survive in a homogeneous environment vs. a heterogeneous environment. Indeed, similar consortia have been grown in homogeneous environments (Hays *et. al.* 2017) and multiple other co-cultures of sucrose-secreting cyanobacteria with other heterotrophs (referenced in the main manuscript). One of the advances we hope that the agent based model can provide is additional insight on the types of influences that a more spatially-defined growth condition imposes, hence in the present work, we sought to further understand the role of spatial proximity.

7. In terms of total biomass (both strains), what conditions maximize it?
   There is a trade-off between the secretion of sucrose ( high concentration of IPTG) and the growth of S. elongatus, so is there an optimal secretion rate?

*S. elongatus* biomass is maximized around 0.1 mM IPTG (Figure 1D). Since *S. elongatus* produces such a small proportion of its biomass as sucrose (as studied here), the populations are heavily dominated by *S. elongatus*, and the answer to the question is as above.

In actuality, there are poorly-understood metabolic exchanges that arise from functions performed by heterotrophs, which appear to provide fitness benefits to cyanobacteria in consortia. We have found that this is true not only for E. coli, but also for a variety of heterotrophic species, including budding yeast, *Bacillus subtilis*, *Pseudomonas putida*, and *Halomonas boleviensis (Hays et al., 2017; Weiss et al., 2016; Fedeson et al., 2019)*. As mentioned in an earlier response (Comment #1 by the same reviewer) we see additional benefits that *S. elongatus* supplies to co-cultured *E. coli* beyond what can be explained by the secreted sucrose. Understanding these emergent beneficial interactions is an active area of study in our lab. Perhaps the slight fitness cost of producing maximum sucrose (1 IPTG of 1 mM) may be offset under real-world conditions (*e.g.*, “positive feedback” from co-cultured heterotrophs). The development of the IBbM in this work is motivated in part so that we can quantitatively predict the impact of known fluxes (*e.g.*, sucrose secretion) so that detection and evaluation of emergent inter-species interactions is possible.

**Reviewer #3:**

1. One major question the author's sought to investigate how well fitness could be predicted. While I believe the authors have explained many of their technical details, I found it difficult - with the ordering of sections in their manuscript - to understand what they meant by "fitness"?

   This is because it wasn't until page 20 (line 375) that the authors state "aggregate colony fitness (total biomass at the end of the simulation".

We apologize for the lack of clarity. We have added additional definitions of fitness to the Abstract (Lines 38-39) and Methods (Line 259).

2. I found it very interesting that the Voronoi tessellation area (aka the growth potential) for a given colony was a poor predictor of fitness (which I'm now interpreting as total colony biomass). Presumably this would not be the case for a different type of microbial species interaction?

For axenic growth, Voronoi tessellation area typically agrees well with colony biomass and its proportion of the total available area. In our case, one reason that it is a poor predictor is that the resources are not evenly distributed in space or time.

3. While the authors did an excellent job of using their framework in a specific context, it would be helpful for the readers if the authors could speak a bit more broadly about how this work might generalize to other types of microbial interactions? Or indeed would all future study require the same detailed fine-tuning the authors performed here?

We thank the reviewer for bringing up this interesting point. Generally, the highest quality and most reliable quantitative results will be had by tuning a model and validating experimentally. While it is possible to simulate other types of interactions without the aforementioned labor and scrutiny, the results would be qualitative, at best. There are several examples of other types of interactions in the original NUFEB paper (*Li et. al.* 2019).

4. When equations end sentences, they should have a period after them. Periods are missing on Equations (12)-(17)

We appreciate the reviewer noticing this error and have corrected the text. We have additionally corrected the equation numbering (12-17 are now 13-18).
